# Supplementary material for: Integrative determination of atomic structure of mutant huntingtin exon 1 fibrils implicated in Huntington disease
Source: Nat Commun. 2024 Dec 30;15:10793. doi: 10.1038/s41467-024-55062-8 (PMC11686214; doi:10.1038/s41467-024-55062-8)
Supplement: Supplementary file 1 — Supplementary Information [file 41467_2024_55062_MOESM1_ESM.pdf]

Supplementary Material: Integrative determination of atomic structure of mutant huntingtin exon 1 fibrils implicated in Huntington disease

Mahdi Bagherpoor Helabad [1,2,3], **Irina Matlahov** [4], **Raj Kumar** [5], **Jan O. Daldrop** [6], **Greeshma Jain** [4], **Markus Weingarth** [5], **Patrick C.A. van der Wel** [4,*] **and Markus S. Miettinen** [1,6,7,8,*]

[1] *Department of Theory and Bio-Systems, Max Planck Institute of Colloids and Interfaces, 14476 Potsdam, Germany*
[2] *Institute for Drug Discovery, Leipzig University Medical Center, 04103 Leipzig, Germany*
[3] *Institute of Chemistry, Martin Luther-University Halle-Wittenberg, 06120 Halle (Saale), Germany*
[4] *Zernike Institute for Advanced Materials, University of Groningen, 9747 AG Groningen, The Netherlands*
[5] *NMR Spectroscopy, Bijvoet Centre for Biomolecular Research, Department of Chemistry, University of Utrecht, 3584 CH Utrecht, The Netherlands*
[6] *Fachbereich Physik, Freie Universität Berlin, 14195 Berlin, Germany*
[7] *Department of Chemistry, University of Bergen, 5007 Bergen, Norway*
[8] *Computational Biology Unit, Department of Informatics, University of Bergen, 5008 Bergen, Norway*

*Corresponding authors:
p.c.a.van.der.wel@rug.nl and
markus.miettinen@iki.fi / markus.miettinen@mpikg.mpg.de

Thursday 28th November, 2024

Supplementary Table 1. System setups in Molecular Dynamics simulations.

System	$x \times y \times z$ [1]	N_{atoms} [2]	$N_{\text{H}_2\text{O}}$ [3]	N_{ion} [4]	N_{pep} [5]	Force field peptide / water
Fully periodic polyQ	3.87×3.16×3.57	5 440	0	0	32	AMBER14SB / –
	3.87×3.16×3.57	5 440	0	0	32	OPLSAA/M / –
	3.87×3.16×3.57	5 440	0	0	32	CHARMM36m / –
PolyQ$_{15}$-*M1*	11.00×11.00×3.81	47 702	9 722	0	56	AMBER14SB / TIP3P
	11.00×11.00×3.81	47 702	9 722	0	56	OPLSAA/M / TIP3P
PolyQ$_{15}$-*M2*	11.00×11.00×3.86	47 780	9 748	0	56	AMBER14SB / TIP3P
	11.00×11.00×3.86	47 750	9 738	0	56	OPLSAA/M / TIP3P
HTTex1-*M2*	38.00×38.00×19.35	2 769 066	840 422	0	140	AMBER14SB / TIP3P

[1] Initial simulation box size (nm^3).
[2] Total number of atoms.
[3] Total number of water molecules.
[4] Total number of (counter / salt) ions.
[5] Total number of peptides.

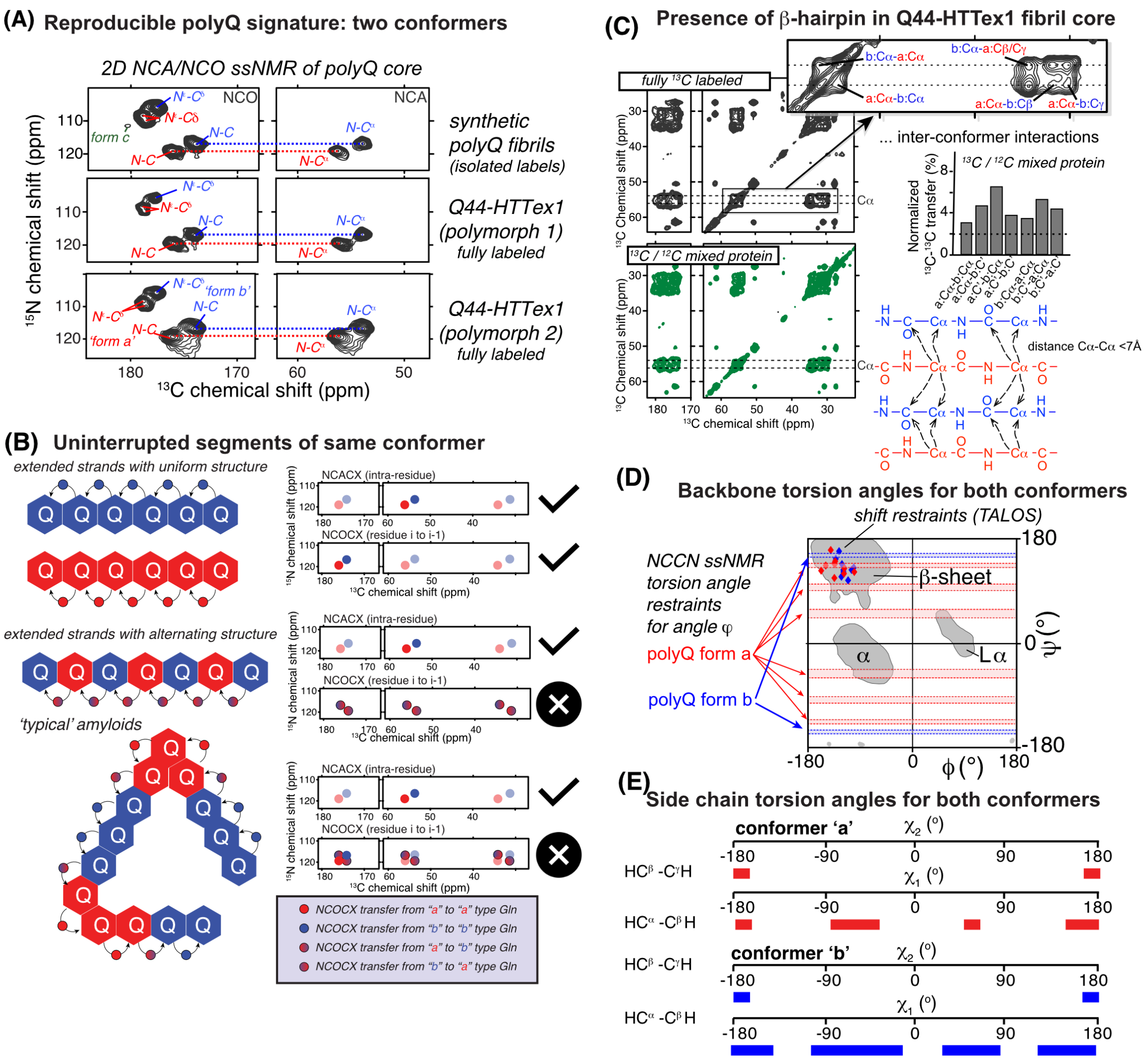

Supplementary Figure 1. Summary of key structural data from prior solid-state NMR studies. (A) 2D spectra showing the nitrogen–carbon correlations in the polyQ backbone and side chains, comparing three different types of fibrils.[1] The left column shows NCO spectra, with correlations for N–C$^{\delta}$ groups in the Gln side chain, and the backbone nitrogen of one Gln with the backbone CO of the preceding amino acid. To the right, NCA spectra show correlations between the backbone N and the C$^{\alpha}$ (intra-residue). The top spectra are for synthetic polyQ peptides with two ^{13}C,^{15}N-labeled Gln residues, and bottom rows show two different types of Q44-HTTex1 fibrils. (B) Peak patterns in the NCA and NCO spectra inform on the connectivity between the two polyQ core conformers. This is illustrated based on three hypothetical models, in which the "a"/"b" conformers form uninterrupted segments (top), alternate in a systematic manner (middle), or form more complex patterns (as in typical amyloid fibril structures). Images on the right illustrate expected peak patterns in NCA/NCO spectra. The NCA spectra (intra-residue peaks) are always unchanged, but the NCO spectra show distinct peak patterns. Only the top model fits the observed data. Figure adapted from Boatz et al. (2020).[2] (C) The presence of a β-hairpin structure in Q44-HTTex1 fibrils was previously demonstrated by NMR comparison of fully ^{13}C-labeled and mixed ^{13}C/^{12}C protein fibrils; see Supplementary Ref. 3 for details. (D) Backbone torsion angles were determined by combination of chemical shift analysis and dipolar recoupling measurements.[3] This figure summarizes these data in a Ramachandran plot. (E) Summary of MAS NMR side chain dihedral angle constraints via dipolar recoupling of CH couplings. Panels C and E adapted with permission from Supplementary Ref. 3. Panels A and D are reused with permission of Portland Press Limited, from "Solid-state nuclear magnetic resonance in the structural study of polyglutamine aggregation", P.C.A. van der Wel, Biochemical Society Transactions Vol. 52(2), 2024; permission conveyed through Copyright Clearance Center, Inc. Readers are referred to the original publications for detailed explanation of symbols and color codes; see also a recent comprehensive review on this topic, Supplementary Ref. 1.

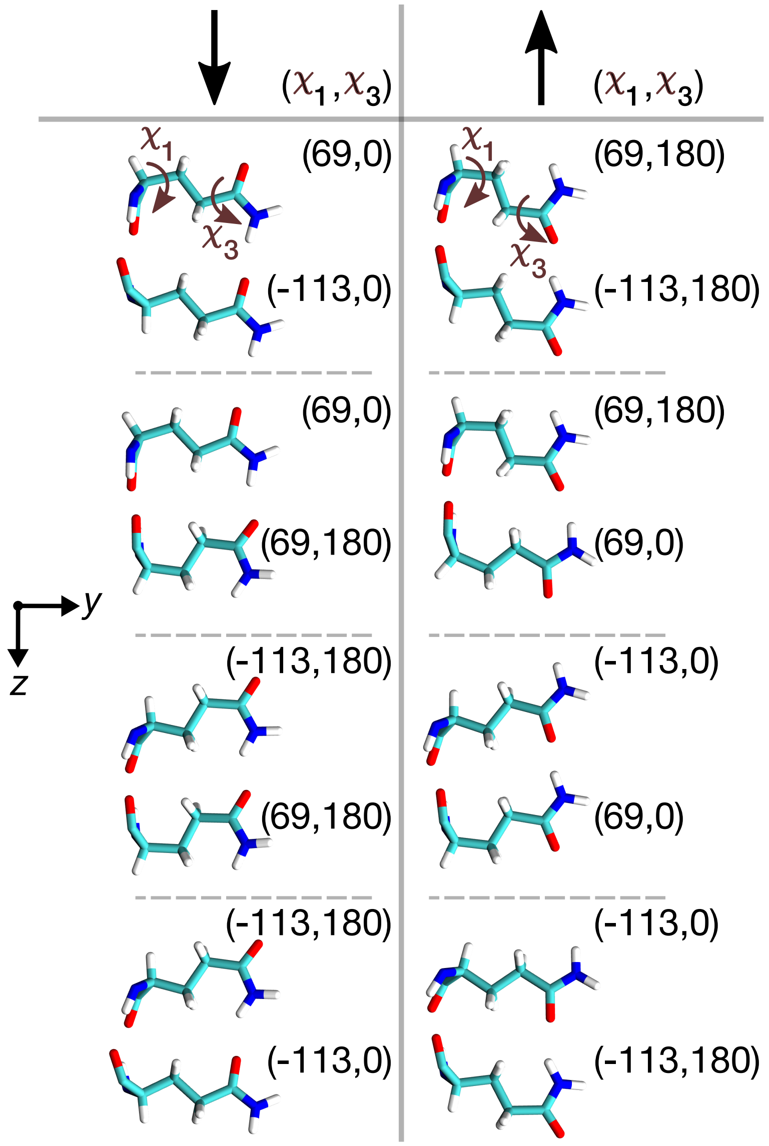

Supplementary Figure 2. Schematic visualization of the eight (χ_1, χ_3) **rotamer classes** of a Gln–Gln pair, in which a side-chain–side-chain hydrogen bonding interaction could take place. The side-chains of the pair are located on neighbouring β-strands of an antiparallel β-sheet inside the polyQ amyloid core. The shown snapshots of these eight unique pairs are taken from the dihedral-restraint-equilibrated conformations, where the dihedral angles (χ_1, χ_3) are restrained to their initial values (listed next to the corresponding sidechain, see also Supplementary Fig. 3). Depending on the H-bond direction, the eight distinct structural classes are categorized into two forms: ↓ and ↑.

(A)

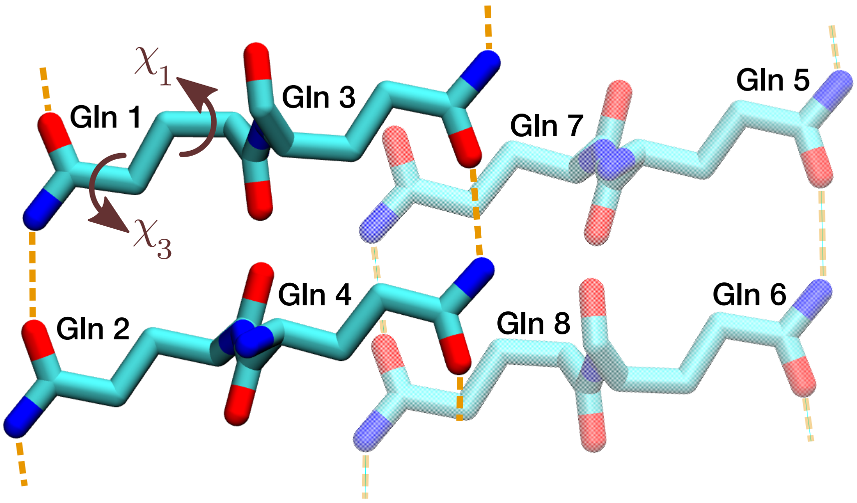

(B)

Systems	Gln 1	Gln 2	Gln 3	Gln 4	Gln 5	Gln 6	Gln 7	Gln 8
**1**	(-113, 180)	(-113, 0)	(-113, 0)	(-113, 180)	(69, 0)	(69, 180)	(69, 180)	(69, 0)
**2**	(-113, 180)	(69, 180)	(-113, 0)	(69, 0)	(-113, 180)	(69, 180)	(-113, 0)	(69, 0)
**3**	(-113, 180)	(69, 180)	(-113, 0)	(69, 0)	(69, 0)	(-113, 0)	(69, 180)	(-113, 180)
**4**	(69, 0)	(-113, 0)	(69, 180)	(-113, 180)	(-113, 180)	(69, 180)	(-113, 0)	(69, 0)
**5**	(69, 0)	(-113, 0)	(69, 180)	(-113, 180)	(69, 0)	(-113, 0)	(69, 180)	(-113, 180)
**6**	(69, 0)	(69, 180)	(69, 180)	(69, 0)	(-113, 180)	(-113, 0)	(-113, 0)	(-113, 180)
**7**	(-113, 0)	(-113, 180)	(-113, 180)	(-113, 0)	(69, 0)	(69, 180)	(69, 180)	(69, 0)
**8**	(-113, 0)	(69, 0)	(-113, 180)	(69, 180)	(-113, 180)	(69, 180)	(-113, 0)	(69, 0)
**9**	(-113, 0)	(69, 0)	(-113, 180)	(69, 180)	(69, 0)	(-113, 0)	(69, 180)	(-113, 180)
**10**	(69, 180)	(-113, 180)	(69, 0)	(-113, 0)	(-113, 180)	(69, 180)	(-113, 0)	(69, 0)
**11**	(69, 180)	(-113, 180)	(69, 0)	(-113, 0)	(69, 0)	(-113, 0)	(69, 180)	(-113, 180)
**12**	(69, 180)	(69, 0)	(69, 0)	(69, 180)	(-113, 180)	(-113, 0)	(-113, 0)	(-113, 180)
**13**	(-113, 180)	(-113, 0)	(-113, 180)	(-113, 0)	(69, 0)	(69, 180)	(69, 180)	(69, 0)
**14**	(-113, 180)	(69, 180)	(-113, 180)	(69, 180)	(-113, 180)	(69, 180)	(-113, 0)	(69, 0)
**15**	(-113, 180)	(69, 180)	(-113, 180)	(69, 180)	(69, 0)	(-113, 0)	(69, 180)	(-113, 180)
**16**	(69, 0)	(-113, 0)	(69, 0)	(-113, 0)	(-113, 180)	(69, 180)	(-113, 0)	(69, 0)
**17**	(69, 0)	(-113, 0)	(69, 0)	(-113, 0)	(69, 0)	(-113, 0)	(69, 180)	(-113, 180)
**18**	(69, 0)	(69, 180)	(69, 0)	(69, 180)	(-113, 180)	(-113, 0)	(-113, 0)	(-113, 180)
**19**	(-113, 180)	(-113, 0)	(-113, 180)	(-113, 0)	(69, 0)	(69, 180)	(69, 0)	(69, 180)
**20**	(-113, 180)	(69, 180)	(-113, 180)	(69, 180)	(-113, 180)	(69, 180)	(-113, 180)	(69, 180)
**21**	(-113, 180)	(69, 180)	(-113, 180)	(69, 180)	(69, 0)	(-113, 0)	(69, 0)	(-113, 0)
**22**	(69, 0)	(-113, 0)	(69, 0)	(-113, 0)	(-113, 180)	(69, 180)	(-113, 180)	(69, 180)
**23 (M1)**	(69, 0)	(-113, 0)	(69, 0)	(-113, 0)	(69, 0)	(-113, 0)	(69, 0)	(-113, 0)
**24**	(69, 0)	(69, 180)	(69, 0)	(69, 180)	(-113, 180)	(-113, 0)	(-113, 180)	(-113, 0)
**25**	(-113, 180)	(-113, 0)	(-113, 180)	(-113, 0)	(69, 180)	(69, 0)	(69, 180)	(69, 0)
**26**	(-113, 180)	(69, 180)	(-113, 180)	(69, 180)	(-113, 0)	(69, 0)	(-113, 0)	(69, 0)
**27**	(-113, 180)	(69, 180)	(-113, 180)	(69, 180)	(69, 180)	(-113, 180)	(69, 180)	(-113, 180)
**28 (M2)**	(69, 0)	(-113, 0)	(69, 0)	(-113, 0)	(-113, 0)	(69, 0)	(-113, 0)	(69, 0)
**29**	(69, 0)	(-113, 0)	(69, 0)	(-113, 0)	(69, 180)	(-113, 180)	(69, 180)	(-113, 180)
**30**	(69, 0)	(69, 180)	(69, 0)	(69, 180)	(-113, 0)	(-113, 180)	(-113, 0)	(-113, 180)

Supplementary Figure 3. (A) **Illustration of the molecular architecture of the eight-glutamine (Gln) building block**, which forms the basis for generating plausible polyQ amyloid core structures. (B) Initial dihedral angle pairs (χ_1, χ_3) of eight Gln residues in a unit cell, for the 30 structurally distinct polyQ amyloid core candidates, with models *M1* and *M2* highlighted in gray. These initial configurations serve as the starting points for molecular dynamics (MD) simulations, aiming to explore the stability of each candidate structure.

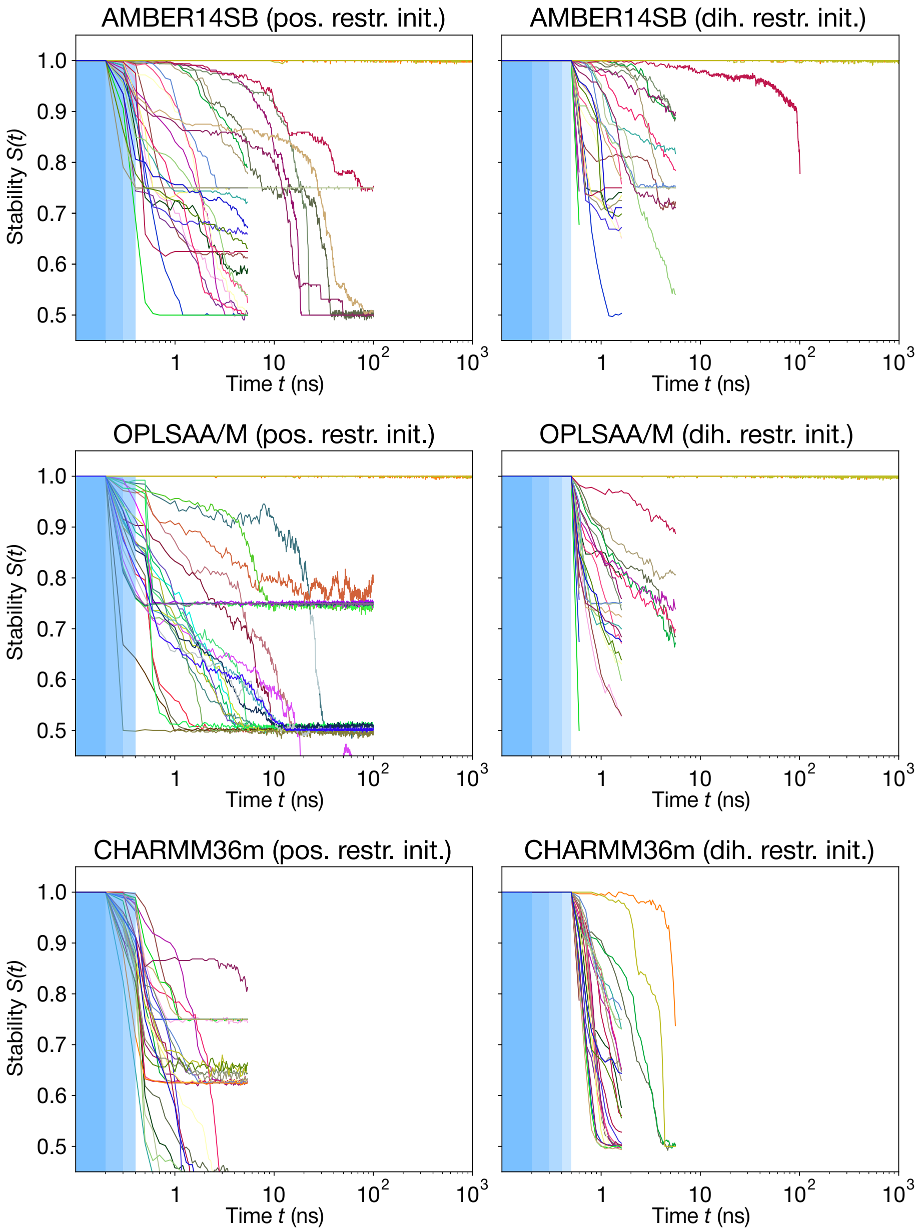

Supplementary Figure 4. Stabilities $S(t)$ (see Eq. (1) in main text Methods) as a function of MD simulation time t for the 30 experimentally-feasible polyQ core candidates (colored lines). The blue-shaded areas indicate the gentle initiating protocol (of applying structural restraints that are gradually removed—such that on the non-shaded area no restraints remain) used to assure that no de-facto-inconsequential incompatibilites between an ideal candidate structure and the force field would lead the structure losing stability during the first few simulation steps. The three blue-shaded areas in the left column indicate three consecutive 100-ps simulations with restraints of 1 000, 500, and 100 kJ/mol/nm^2 on heavy (non-H) atom positions. The four blue-shaded areas in the right column indicate restraints of 1 000, 500, 250, and 100 kJ/mol/rad^2 on χ_1 and χ_3 dihedral angles. The top left panel shows the first 1 µs of main text Fig. 2B.

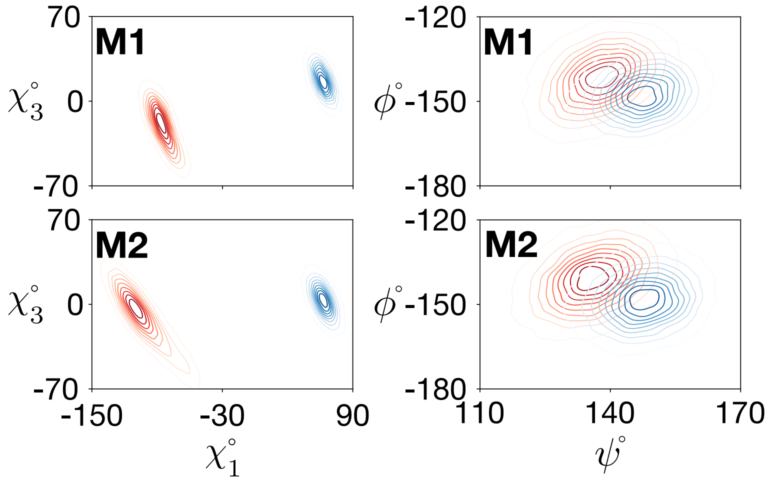

Supplementary Figure 5. The side-chain χ_1–χ_3 (left panels) and backbone ψ–ϕ (right) dihedral angle distributions of conformers "a" (red) and "b" (blue) for the final models *M1* (top) and *M2* (bottom). Calculations were performed using the OPLSAA/M force field; for Amber14SB see main text Fig. 2D.

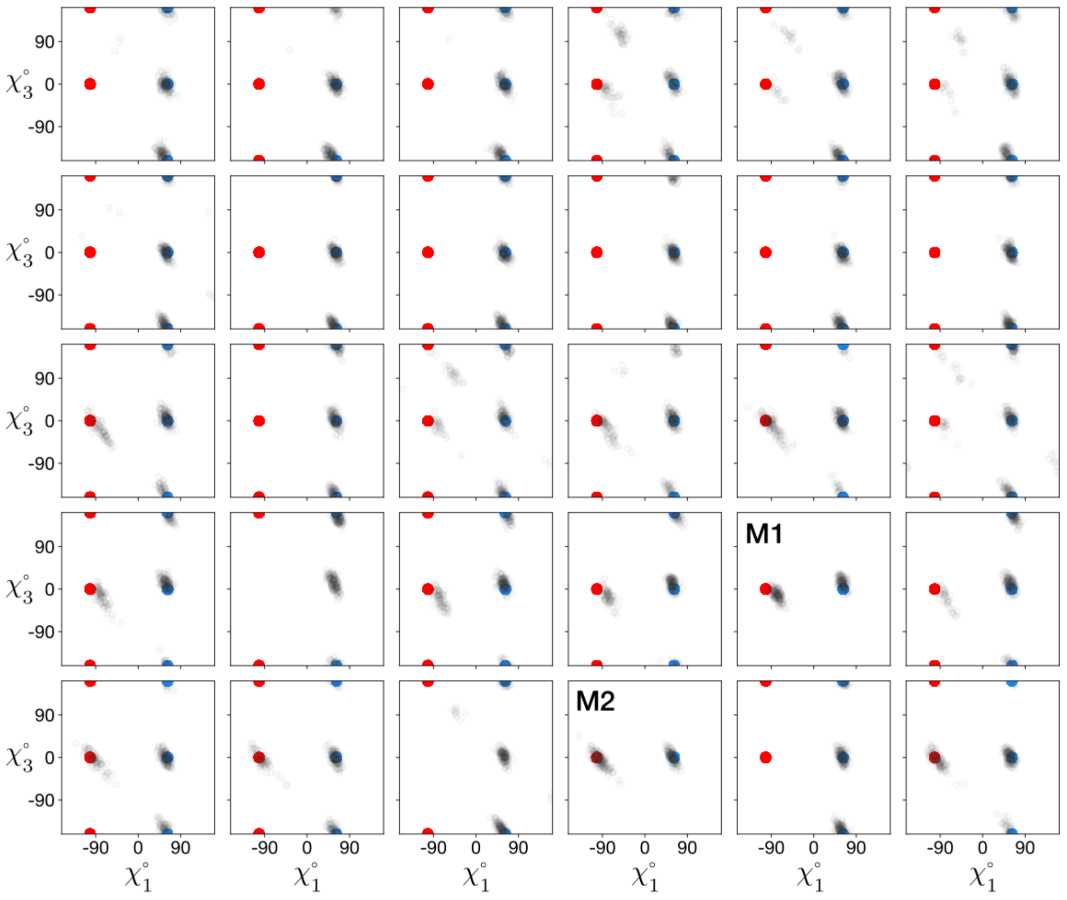

Supplementary Figure 6. Dihedral angle distributions of χ_1 and χ_3 for 30 structurally distinct polyQ amyloid core candidates. The gray distributions represent the dihedral angles of the last frames from the 30 MD simulations. The red and blue points indicate the initial χ_1 and χ_3 dihedral angles for the two populated conformers "a" and "b", respectively.

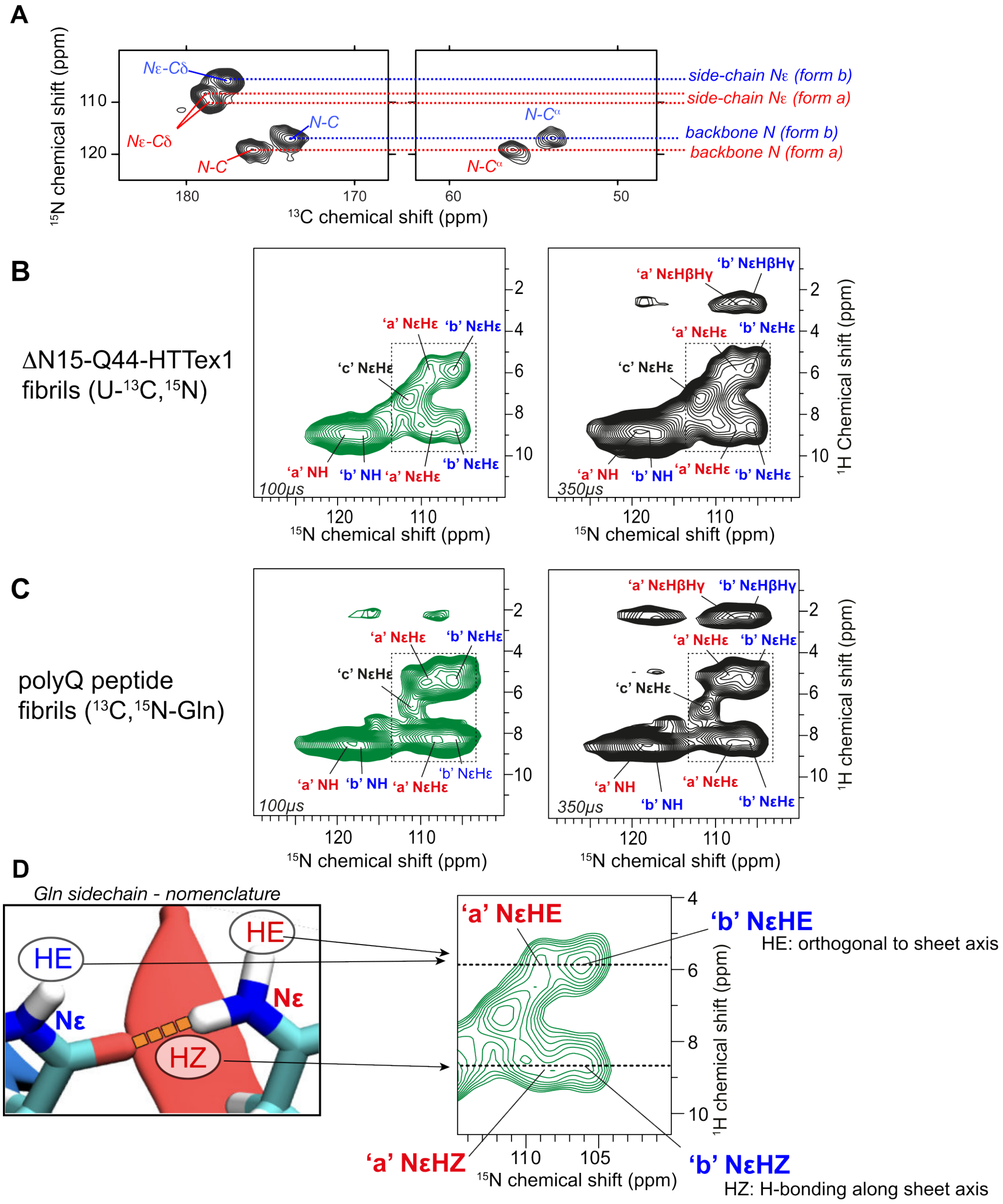

Supplementary Figure 7. MAS NMR assignment spectra for the Gln side chain protons in the polyQ core. (A) NCA and NCO spectra reproduced from Supplementary Fig. 1A (top), showing the nitrogen–carbon correlations from polyQ core residues. The "a" and "b" conformers are color-coded red and blue, respectively. Dashed lines mark the ^{15}N chemical shifts that we can thus assign, for the backbone (near 120 ppm) and side chain (near 105–110 ppm). (B) ^{15}N-detected 2D HN correlation spectra for fully labeled ΔN15-Q44-HTTex1 fibrils, obtained by trypsin cleavage. The ^{15}N assignments from panel (A) allow the identification and assignment of the corresponding peaks. The spectrum at the left was recorded with 0.1 ms contact time (showing directly bonded correlations); the right one 0.35 ms contact time (adding some longer range peaks involving nearby carbon-attached protons). (C) ^{15}N-detected 2D HN correlation spectra for polyQ peptide fibrils ($K_2Q_{11}pGQ_{11}K_2$; see Methods section) outfitted only with labeled Gln residues (thus avoiding signals from non-Gln residues). Comparison of the panels B and C shows that the polyQ peptide has the same spectroscopic (and thus structural) features. The close correspondence shows also that the observed peaks for the protein are indeed explained by the polyQ core. (D) H_E and H_Z nomenclature[4] for Gln-ladder hydrogen bonding (see also main text), illustrated and mapped onto the polyQ core signals from panel B (right).

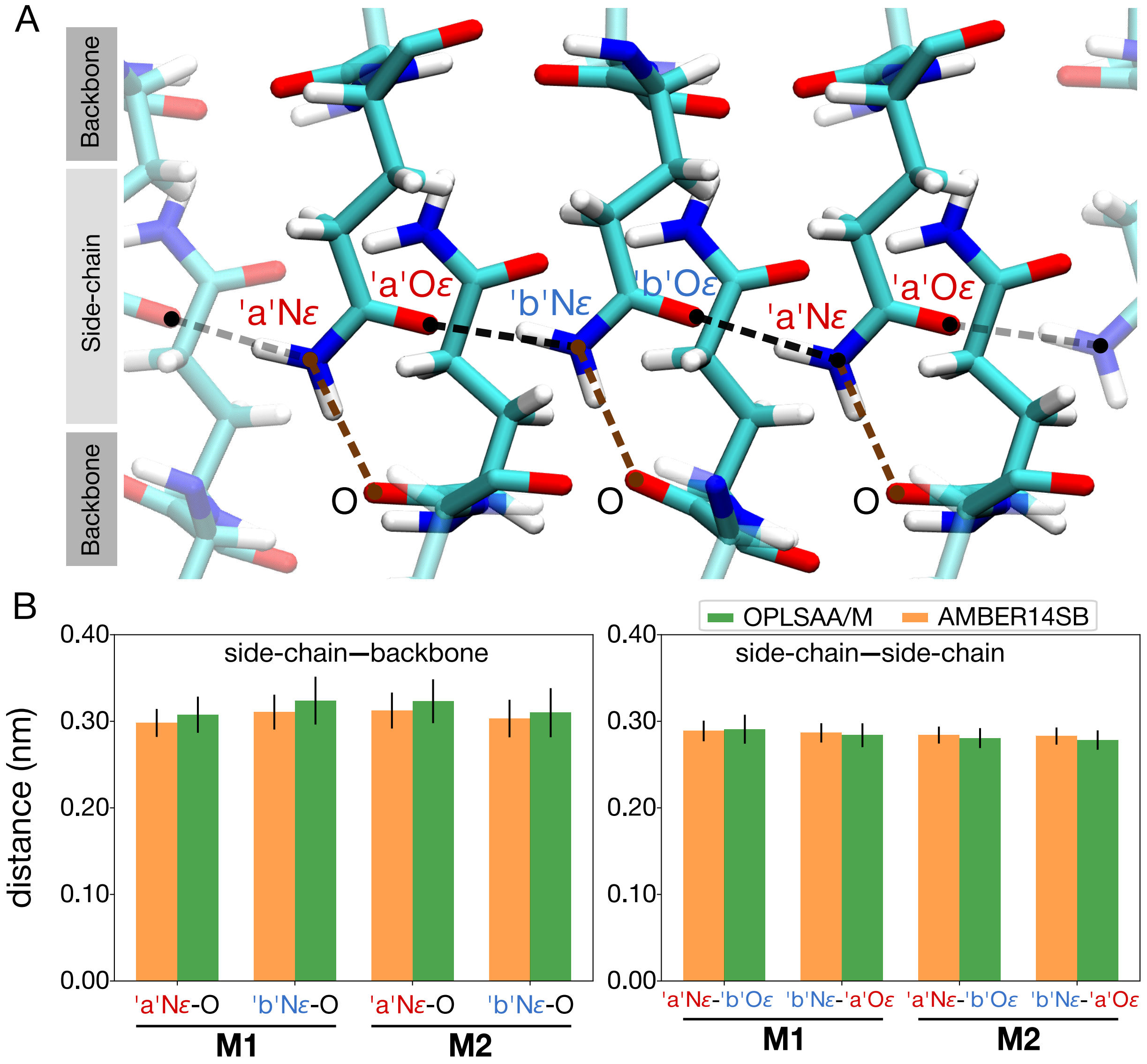

Supplementary Figure 8. (A) Spatial arrangement of the side-chain and backbone atoms in the polyQ amyloid core model *M2*. Brown dashed lines represent the distances between the N_ϵ atoms of the "a" and "b" types of Gln residues and their nearest backbone O atoms, reflecting the H_E nomenclature discussed in the main text.[4] Black dashed lines show the N_ϵ–O_ϵ distances between side-chains, reflecting the H_Z amide hydrogens. (B) Average side-chain–backbone distances (left panel), i.e., the pairwise distances between the N_ϵ atoms of the "a" and "b" types of Gln residues and their closest backbone O atoms (indicative of H_E intersheet H-bonding). Average side-chain–side-chain distances (right panel), i.e., the N_ϵ–O_ϵ distances between the side chains, either from "a" to "b" or from "b" to "a" (indicative of H_Z intrasheet H-bonding). All 160 individual pairwise distances of each of these four distance types were calculated from 5 000 frames (taken from the whole 1-µs simulation at 200 ps intervals); bar height shows mean, error bars the standard deviation ($n = 160 \times 5000$). Data are shown for both the *M1* and *M2* models, and using two force fields: OPLSAA/M (green) and AMBER14SB (orange).

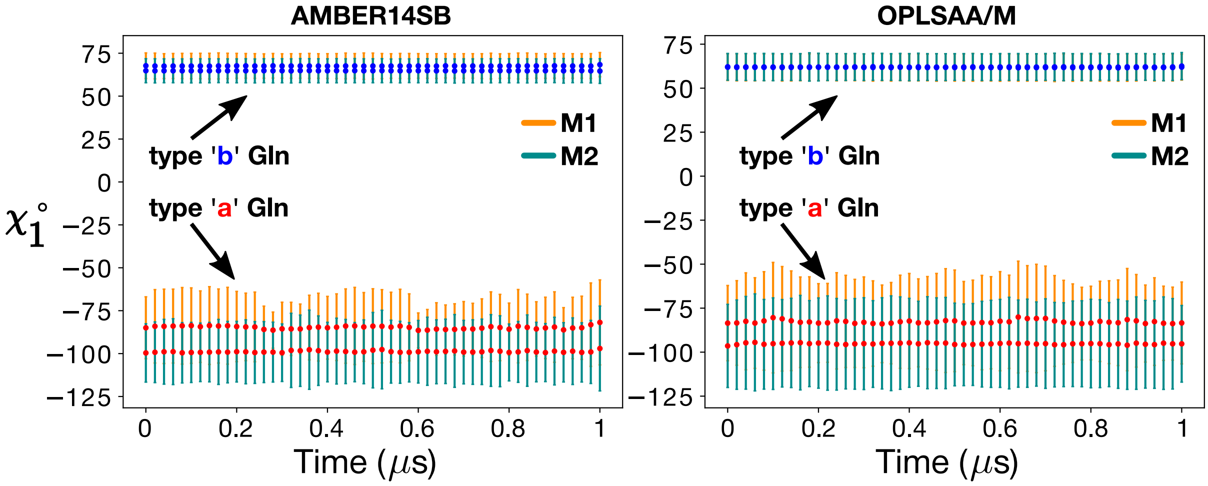

Supplementary Figure 9. Both polyQ$_{15}$ models *M1* (orange) and *M2* (green) remained stable throughout 1-μs MD simulations in both the AMBER14SB (left) and OPLSAA/M (right) force fields. Shown are the mean χ_1 dihedral angles in type "a" (red) and "b" (blue) Gln side-chains, calculated over consecutive 20-ns windows. In each 20-ns window, calculations were performed over frames taken at 200-ps intervals, including 300 type "a" and 300 type "b" Gln residues (that is: all Gln residues of a given type except those in the two water-exposed β-sheets); error bars show standard deviation ($n = 300 \times 100$).

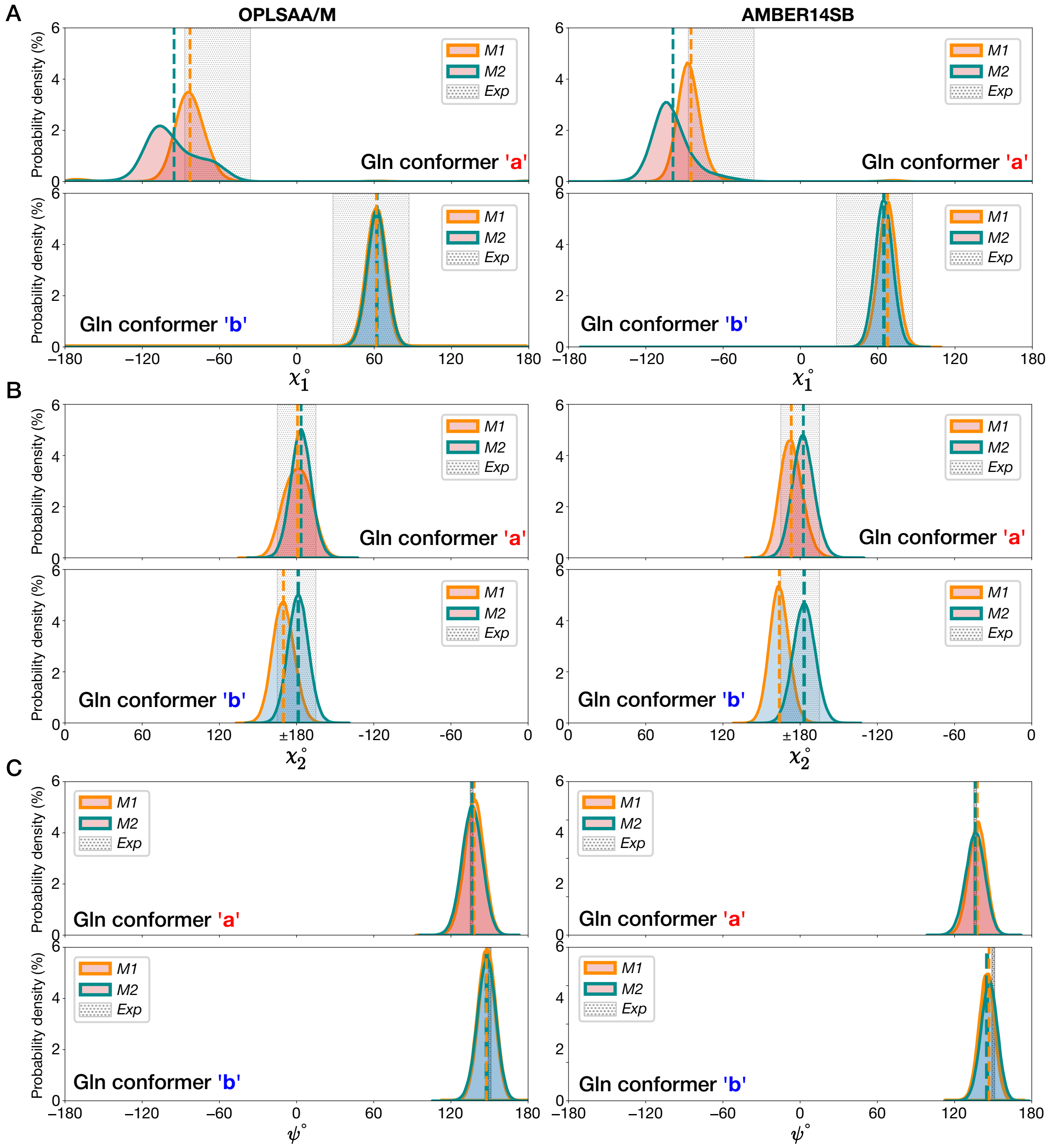

Supplementary Figure 10. Distributions of the (A) χ_1, (B) χ_2, and (C) ψ dihedral angles in the *M1* (orange lines) and *M2* (green lines) models of the $D_2Q_{15}K_2$ fibril obtained from 1-μs MD simulations using the OPLSAA/M (left) and AMBER14SB (right) force fields. The upper panels show the type "a" conformers (red-shaded distributions) and the lower panels the type "b" (blue-shaded). The gray-shaded regions represent the ssNMR-informed constraints. The dashed vertical lines depict the mean values of the corresponding dihedral angles.

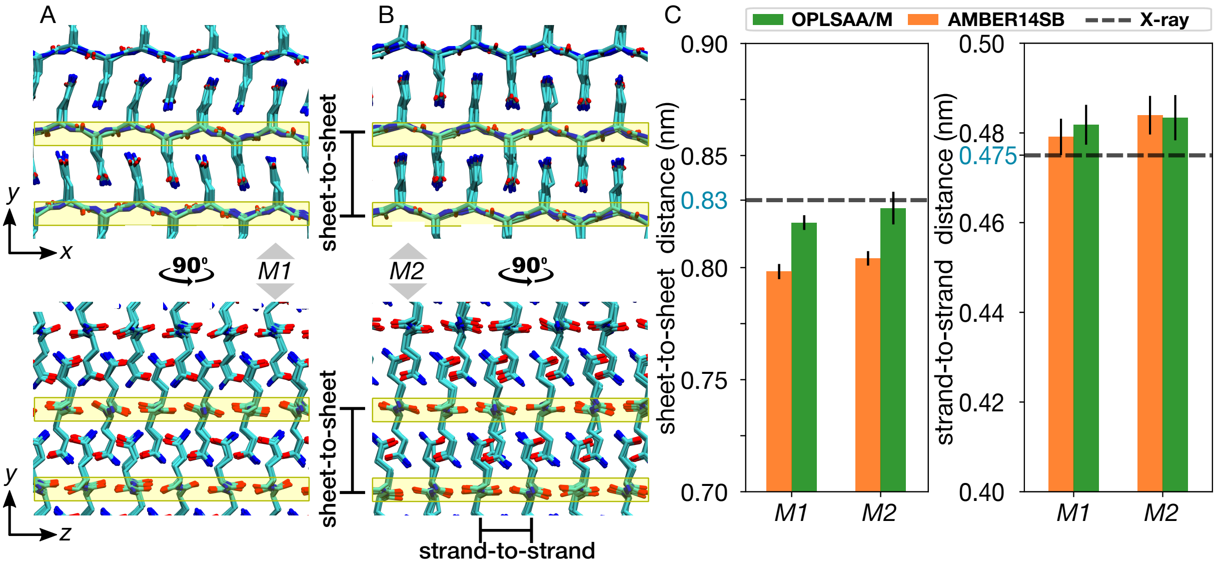

Supplementary Figure 11. Illustration of the structural differences between the (A) *M1* and (B) *M2* models, visualized through views along the fibril axis z (top panels) and along the β-strand direction x (bottom panels). The yellow boxes highlight backbone atoms. (C) Sheet-to-sheet (left panel) and strand-to-strand (right panel) distances calculated for both the *M1* and *M2* models of polyQ$_{15}$ using the AMBER14SB (orange) and OPLSAA/M (green) force fields. The dashed black horizontal lines corresponds to data obtained from X-ray experiments.[5,6] Calculations were performed over 1-µs MD trajectories, analyzing frames at 200-ps intervals; as the two water-exposed β-sheets were excluded, each frame provided four sheet-to-sheet and 40 strand-to-strand distances; error bars represent standard deviation ($n = 4 \times 5\,000$ for sheet-to-sheet and $40 \times 5\,000$ for strand-to-strand distances).

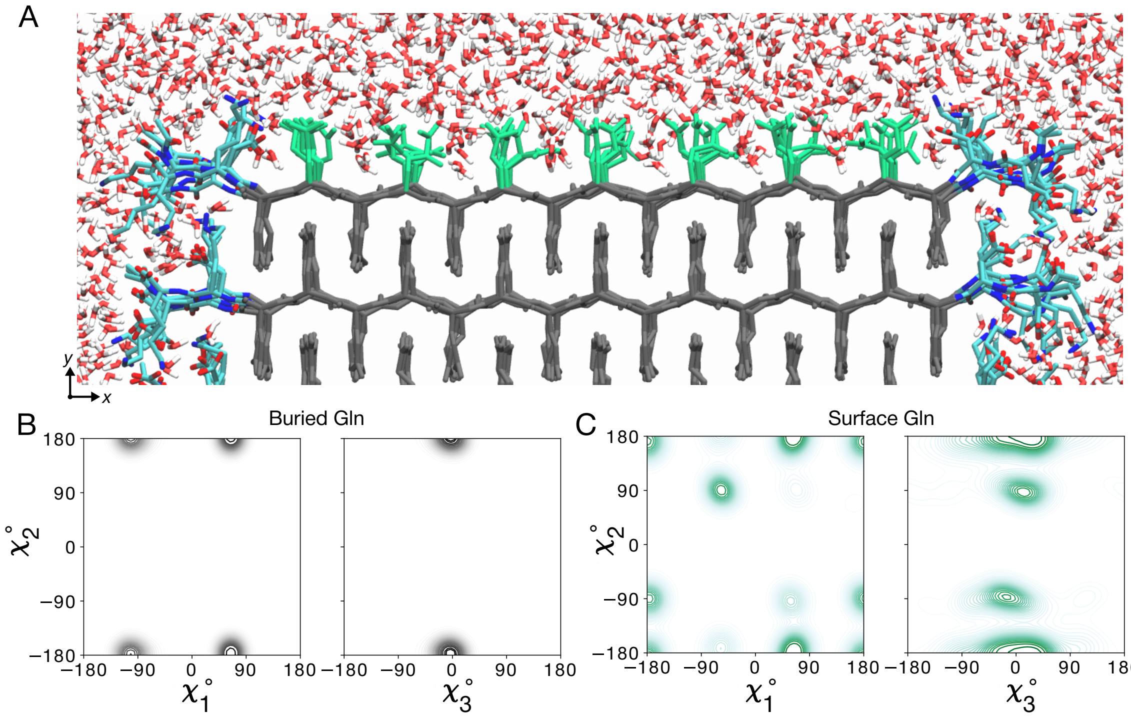

Supplementary Figure 12. Water-facing surface of polyQ amyloid. (A) Atomistic MD snapshot of the $D_2Q_{15}K_2$ peptide fibril's polyQ surface in contact with water. Exposed and buried Gln residues are colored green and gray, respectively. Note how the Gln side-chains internal to the (model *M2*) amyloid core are well-ordered, while the water-facing side-chains display more mobility. (B) Side-chain dihedral angle distributions for the buried Gln residues and (C) for the Gln residues on the fibril surface (Amber14SB[7]). The surface-facing residues show more disorder, but are nonetheless constrained to eight varyingly prominent specific rotamer states.

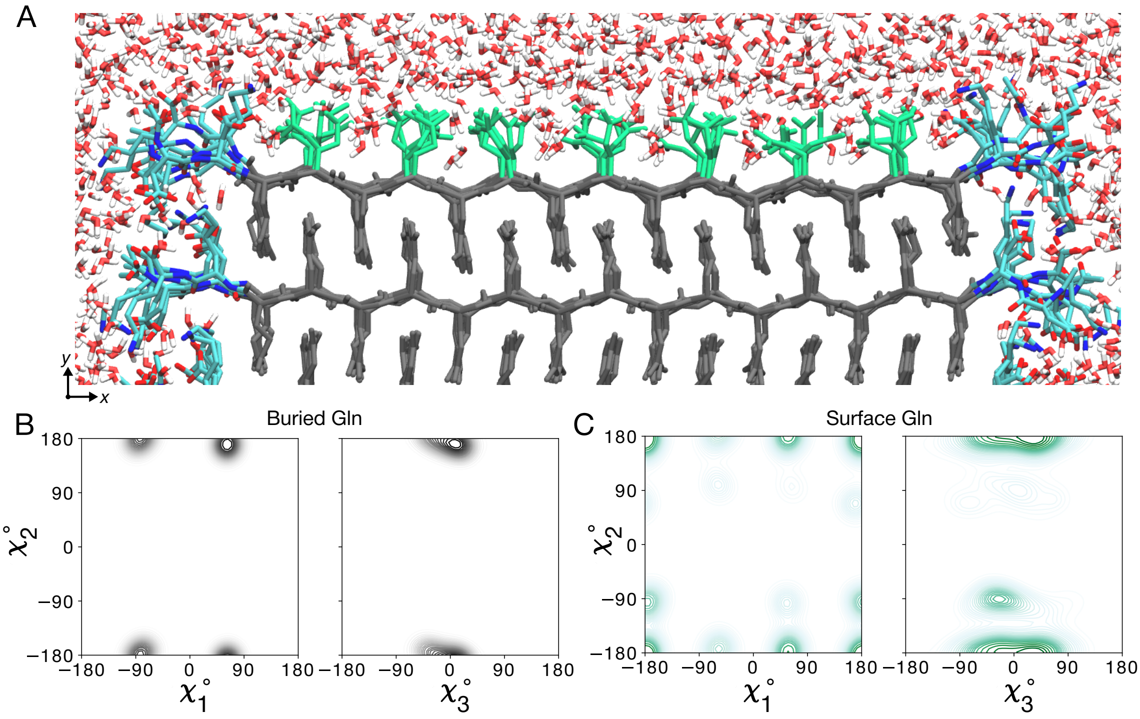

Supplementary Figure 13. Water-facing surface of polyQ amyloid. (A) Atomistic MD snapshot of the $D_2Q_{15}K_2$ peptide fibril's polyQ surface in contact with water. Exposed and buried Gln residues are colored green and gray, respectively. Note how the Gln side-chains internal to the (model *M1*) amyloid core are well-ordered, while the water-facing side-chains display more mobility. (B) Side-chain dihedral angle distributions for the buried Gln residues and (C) for the Gln residues on the fibril surface (OPLSAA/M[8]). The surface-facing residues show more disorder, but are nonetheless constrained to eight varyingly prominent specific rotamer states.

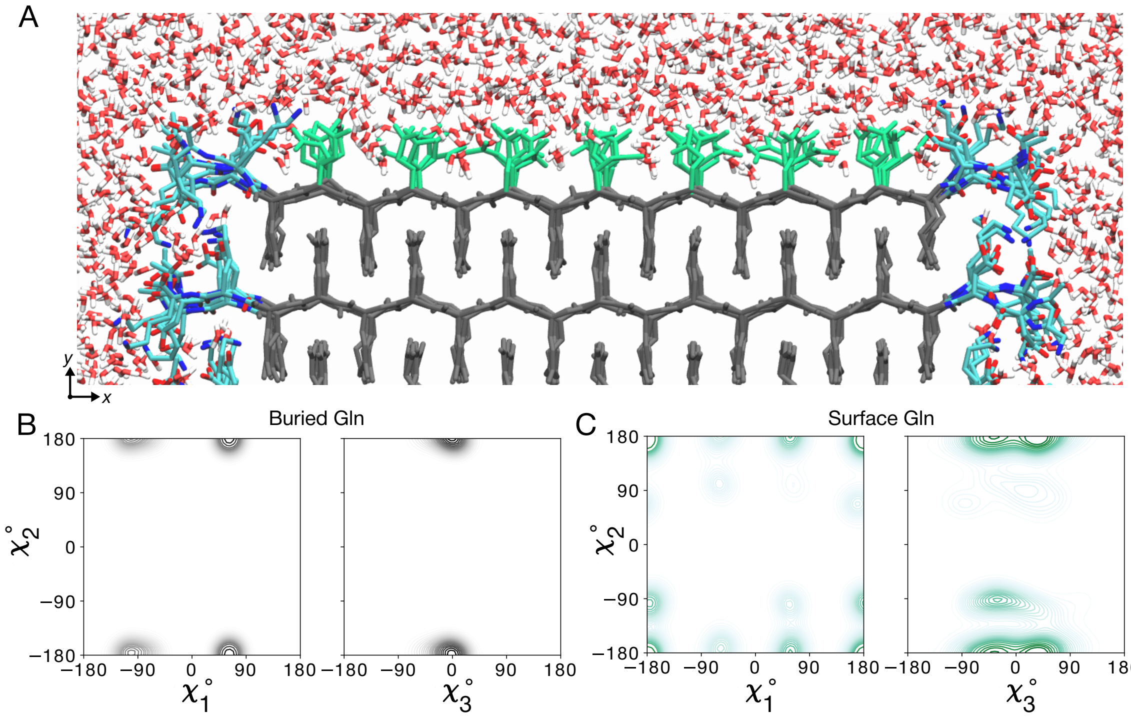

Supplementary Figure 14. Water-facing surface of polyQ amyloid. (A) Atomistic MD snapshot of the $D_2Q_{15}K_2$ peptide fibril's polyQ surface in contact with water. Exposed and buried Gln residues are colored green and gray, respectively. Note how the Gln side-chains internal to the (model *M2*) amyloid core are well-ordered, while the water-facing side-chains display more mobility. (B) Side-chain dihedral angle distributions for the buried Gln residues and (C) for the Gln residues on the fibril surface (OPLSAA/M[8]). The surface-facing residues show more disorder, but are nonetheless constrained to eight varyingly prominent specific rotamer states.

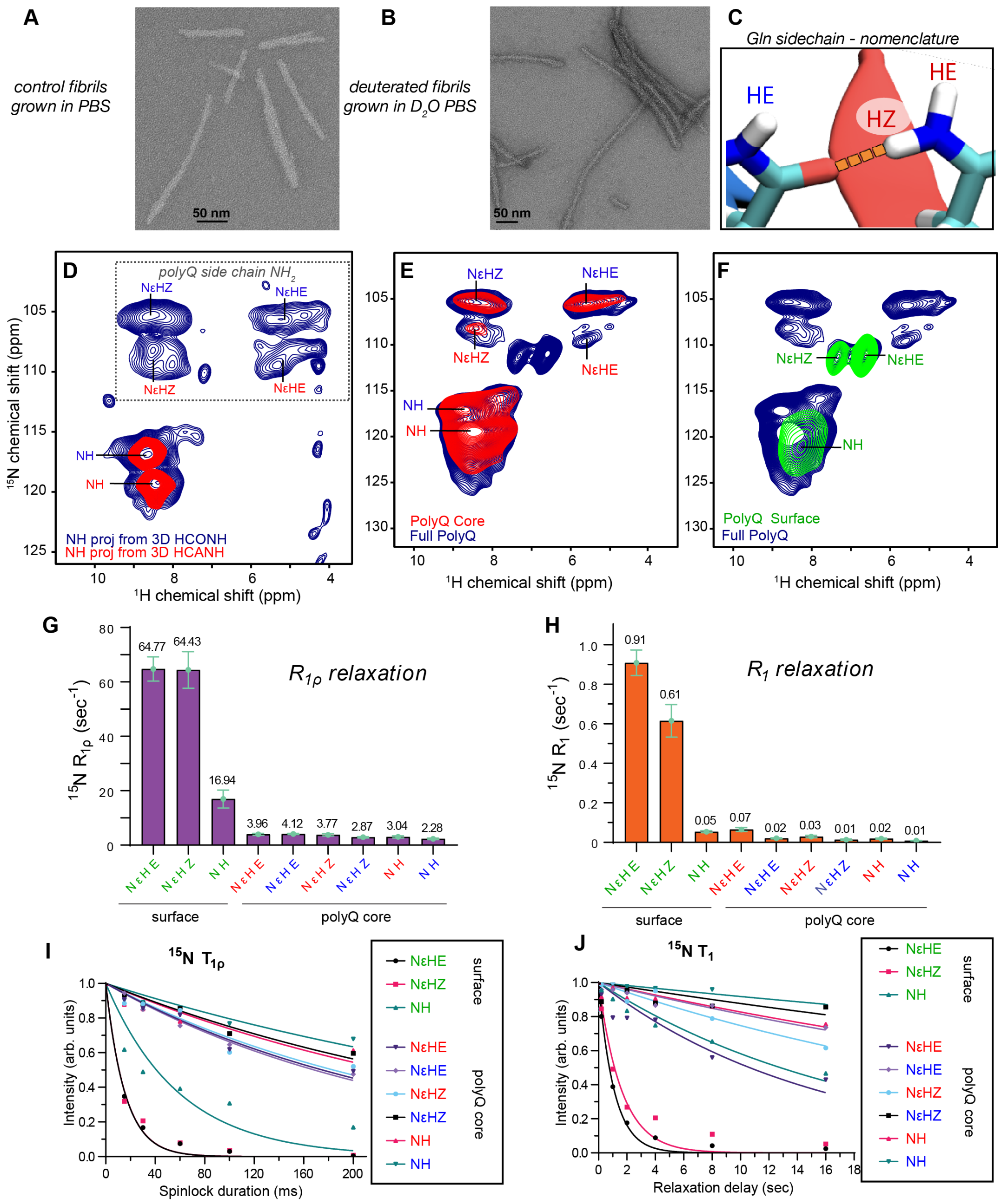

Supplementary Figure 15. MAS NMR of core and surface polyQ residues of Q44-HTTex1 fibrils submitted to HDX. (A,B) Negative stain TEM of Q44-HTTex1 fibrils prepared in regular (protonated) PBS buffer (A), or in PBS buffer made with D_2O (B). The EM data are representative results from 20 micrographs recorded on a single prepared EM grid for each sample. (C) Illustration of the H_E vs H_Z nomenclature used for the NH_2 hydrogens in the Gln ladders (see also main text). (D) 2D projections of 3D ^{1}H-detected HCONH and HCANH 3D spectra for fully protonated Q44-HTTex1 fibrils. These spectra can be compared to the ^{15}N-detected spectra in Supplementary Fig. 7, and are predominantly derived from the polyQ amyloid core of these HTTex1 fibrils. (E) Overlay of ^{1}H-detected 2D HN spectra for fully protonated (blue) and surface-deuterated HTTex1 fibrils. These data are obtained for fibrils grown in regular protonated PBS, which were washed with deuterated PBS prior to MAS NMR analysis. Thus, exchangeable protons should disappear, but protected amide groups are visible (i.e., the polyQ core internals). (F) Analogous data for fibrils grown in deuterated PBS and washed with regular PBS prior to NMR analysis, showing only exchangable amide signals that we attribute to the polyQ surface. (G–H) Bar graphs of ^{15}N $T_{1\rho}$ and T_1 relaxation data for core and surface signals, showing the fitted value with error bars indicating the standard error of the fit. (I–J) Relaxation decay curves related to panels G–H. Data measured at 60 kHz MAS, 700 MHz, 253 K setpoint temperature.

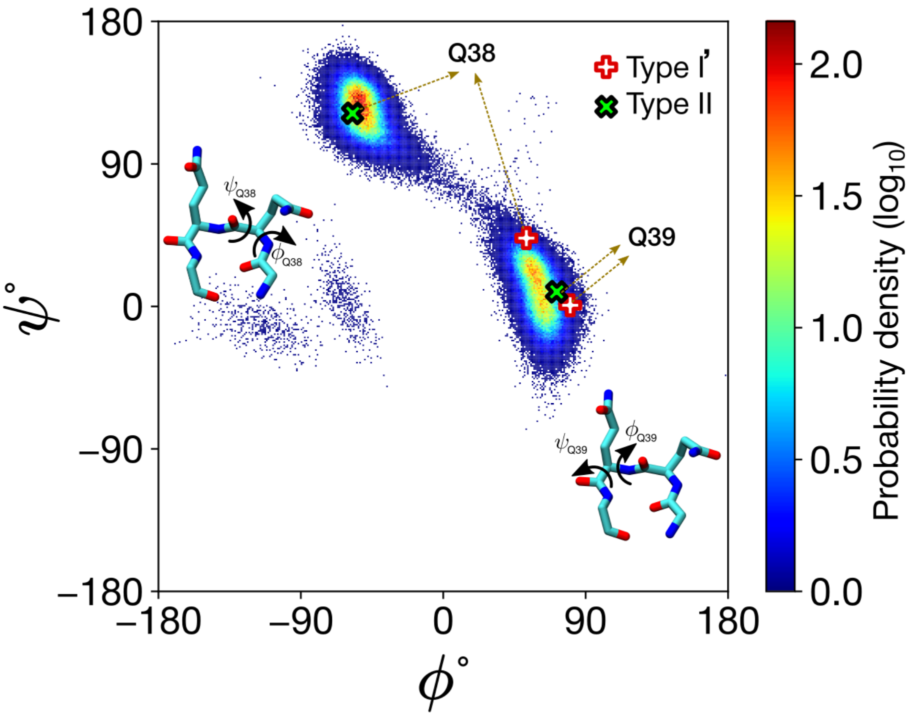

Supplementary Figure 16. Ramachandran plot for the β-turn residues (Q38 and Q39) in the Q44-HTTex1 polyQ amyloid core. The distribution was obtained over the last 1 µs of the 5-µs MD simulation. The β-turn was initially prepared as a type I' conformer (whose canonical dihedrals are indicated with the red/white plus-signs); however, during the simulation it transitioned to the type II (black/green crosses).

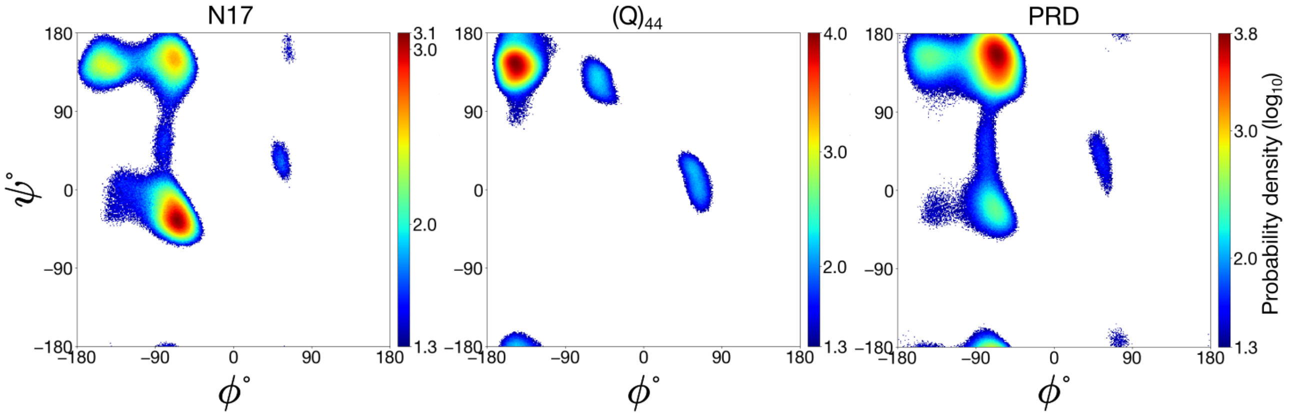

Supplementary Figure 17. Ramachandran plots of the N17, Q_{44}, and PRD domains elucidate the characteristic secondary structures present in these three disparate domains of the HTTex1 protein: α-helical, β-sheet, and PPII-helical, respectively. The distributions were obtained over the last 1 µs of the 5-µs MD simulation. For clarity, $\log_{10}$(probability density) values below 1.3, characterized primarily by noise, have been excluded.

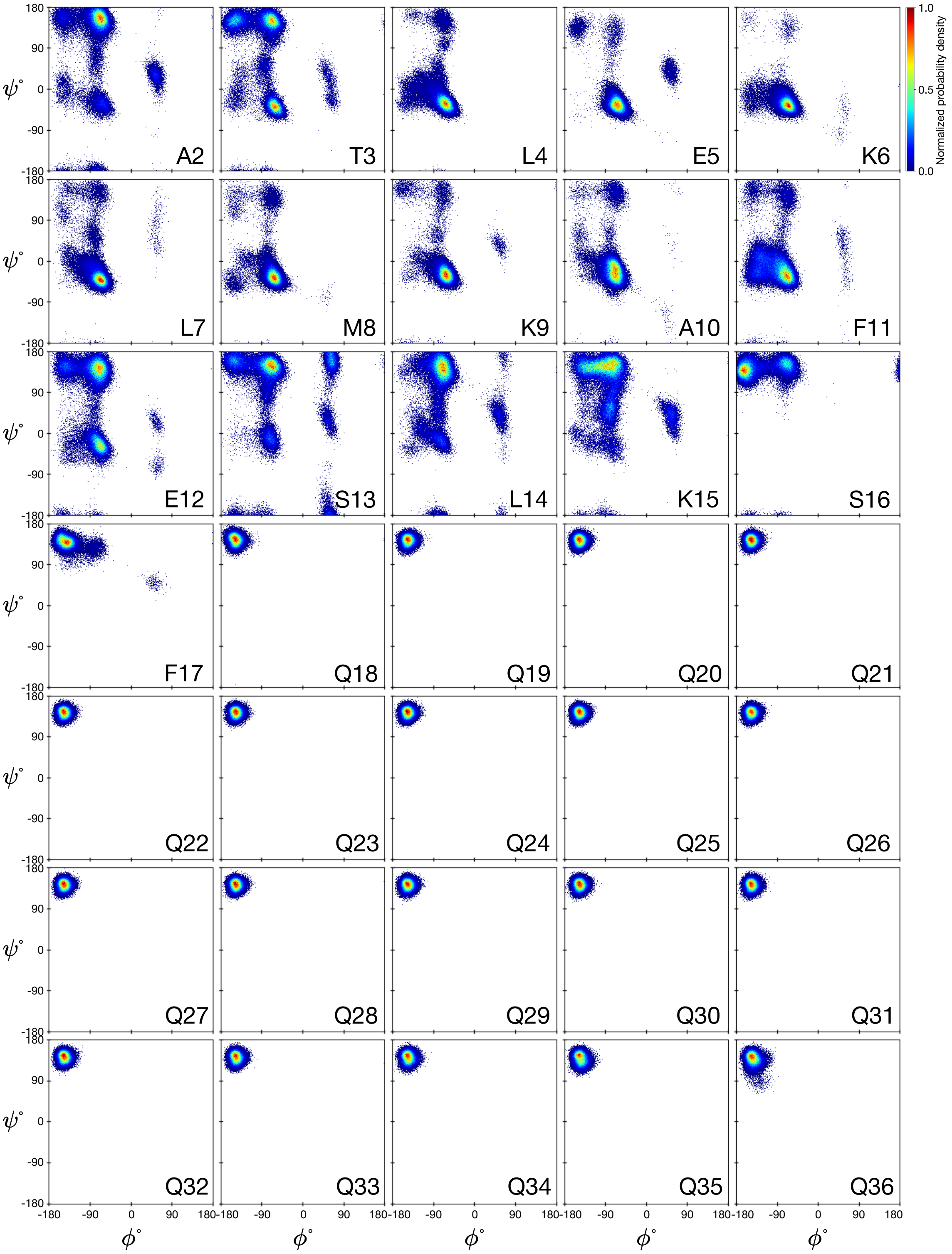

Supplementary Figure 18. Ramachandran plots for residues 2–36 in the Q44-HTTex1 fibril. The plots showcase the conformational space explored by each individual residue, revealing variations in backbone torsion angles across the protein structure. The colorbar is calibrated such that a normalized value of 1 corresponds to the bin with the highest frequency among all protein residues. Each bin spans one degree in both ϕ and ψ. The distributions were obtained over the last 1 µs of the 5-µs MD simulation.

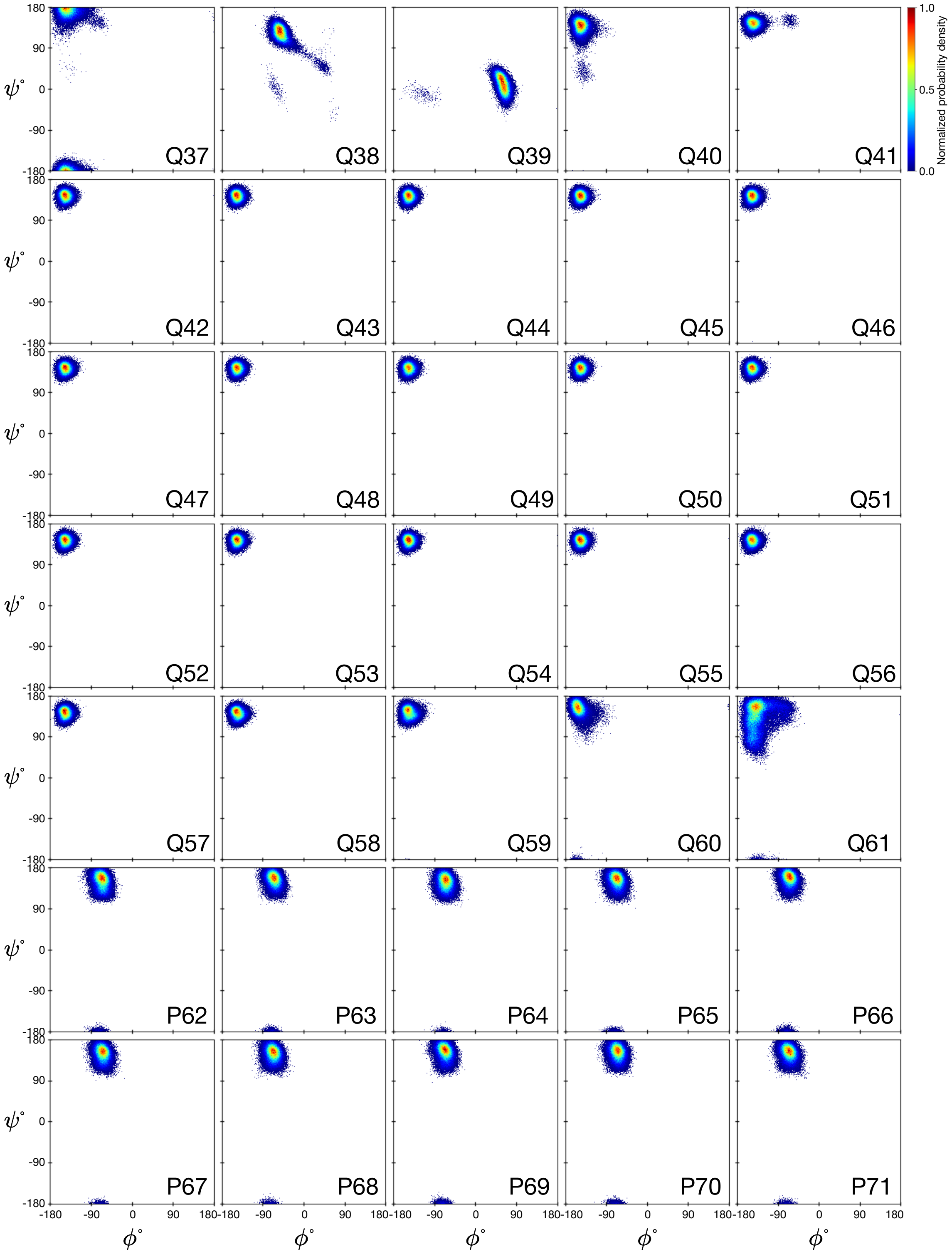

Supplementary Figure 19. Ramachandran plots for residues 37–71 in the Q44-HTTex1 fibril. The plots showcase the conformational space explored by each individual residue, revealing variations in backbone torsion angles across the protein structure. The colorbar is calibrated such that a normalized value of 1 corresponds to the bin with the highest frequency among all protein residues. Each bin spans one degree in both ϕ and ψ. The distributions were obtained over the last 1 µs of the 5-µs MD simulation.

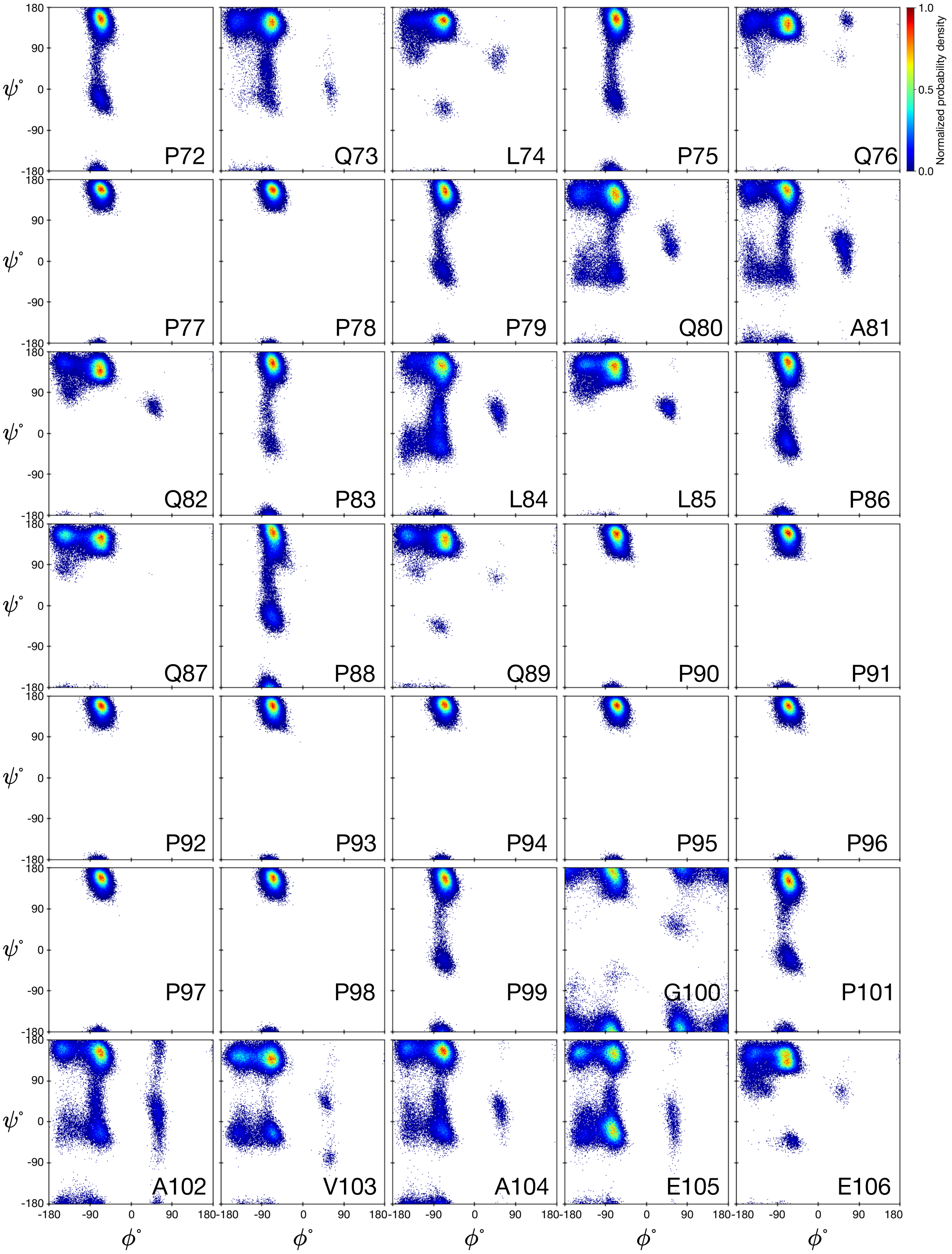

Supplementary Figure 20. Ramachandran plots for residues 72–106 in the Q44-HTTex1 fibril. The plots showcase the conformational space explored by each individual residue, revealing variations in backbone torsion angles across the protein structure. The colorbar is calibrated such that a normalized value of 1 corresponds to the bin with the highest frequency among all protein residues. Each bin spans one degree in both ϕ and ψ. The distributions were obtained over the last 1 µs of the 5-µs MD simulation.

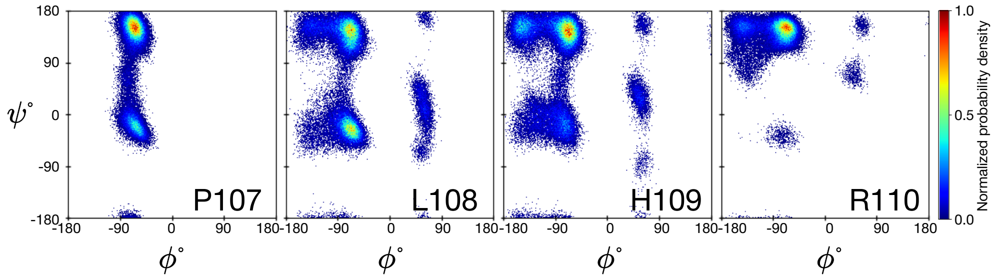

Supplementary Figure 21. Ramachandran plots for residues 107–110 in the Q44-HTTex1 fibril. The plots showcase the conformational space explored by each individual residue, revealing variations in backbone torsion angles across the protein structure. The colorbar is calibrated such that a normalized value of 1 corresponds to the bin with the highest frequency among all protein residues. Each bin spans one degree in both ϕ and ψ. The distributions were obtained over the last 1 µs of the 5-µs MD simulation.

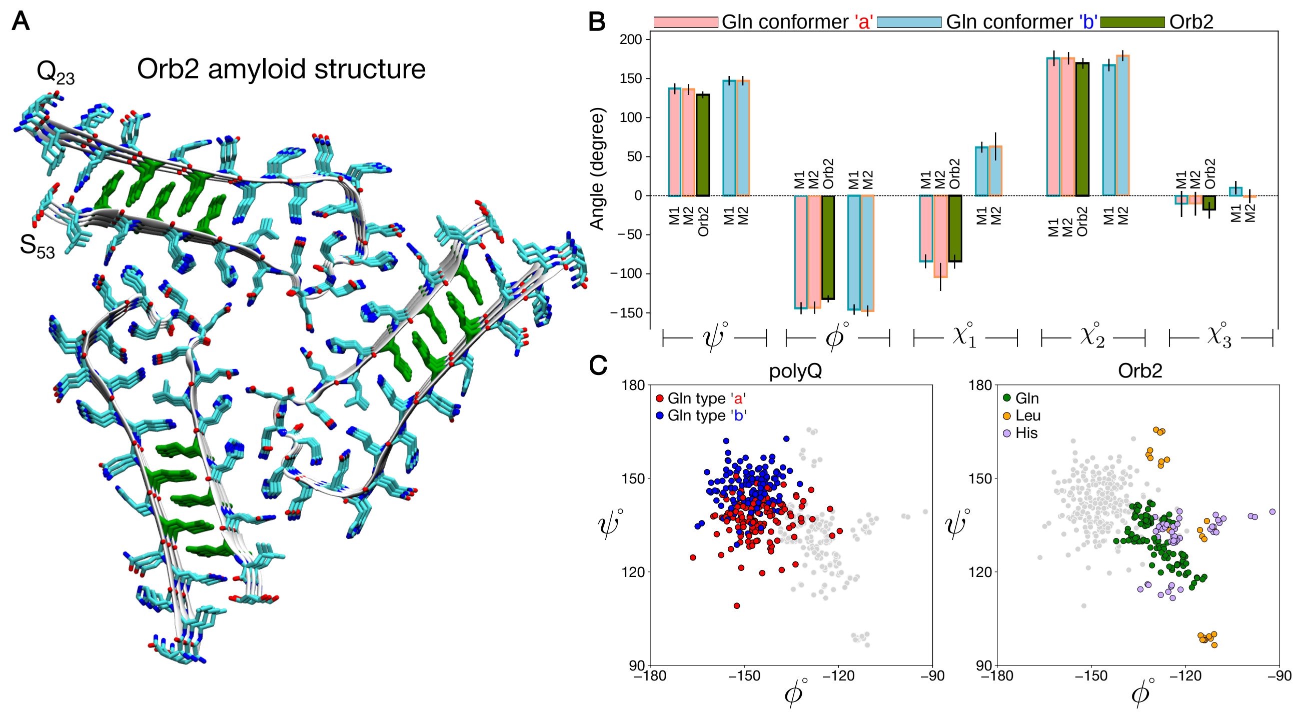

Supplementary Figure 22. Comparison of polyQ amyloid fold to that of glutamine-rich functional amyloid Orb2. (A) Cryo-EM structure of the functional amyloid Orb2, showing its glutamine-rich amyloid core (PDB ID: 6VPS).[9] Gln residues in the amyloid core are shown in green, where they form an interdigitated zipper sheet–sheet interface just like polyQ amyloid . However, in contrast to polyQ, this protein adopts the more common parallel and in-register β-sheet structure that allows identical residues to stack atop each other. Another noteworthy difference to polyQ is that the polypeptide chain in Orb2 forms a β-arcade,[10] stabilized by intermolecular backbone hydrogen bonds, whereas β-hairpin-containing sheets for expanded polyQ are also stabilized by intra-molecular hydrogen bonding. (B) Depicted are the mean ψ, ϕ, χ_1, χ_2, and χ_3 dihedral angles of conformers “a” (displayed in light red) and “b” (light blue) within the *M1* and *M2* models, as well as of the Gln residues within the core domain (specifically residues 28, 30, 47, 49, and 51) of the Orb2 amyloid structure[9] (green). For *M1* and *M2*, 128 deeply buried Gln residues from the final frames of the 1-µs polyQ$_{15}$ MD simulations were used. Error bars represent standard deviation over residues ($n = 45$ for Orb2, and $n = 128$ for both *M1* and *M2*). (C) β-sheet regions of Ramachandran plots, comparing polyQ Gln residues from model *M1* (left) and the Orb2 residues located within the β-strand regions (residues 24–33 and 45–52) of the Orb2 amyloid structure (right). Each plot shows both polyQ and Orb2; however, in the plot representing polyQ *M1*, the Orb2 residues are shown as grey dots, and conversely, in the plot for Orb2, the polyQ residues are marked in grey.

Supplementary References

[1] Van der Wel PC. Solid-state nuclear magnetic resonance in the structural study of polyglutamine aggregation. *Biochemical Society Transactions* **52** (2024) 719–731. doi:10.1042/BST20230731.

[2] Boatz JC, Piretra T, Lasorsa A, Matlahov I, Conway JF, van der Wel PC. Protofilament structure and supramolecular polymorphism of aggregated mutant huntingtin exon 1. *Journal of molecular biology* **432** (2020) 4722–4744.

[3] Hoop CL, Lin HK, Kar K, Magyarfalvi G, Lamley JM, Boatz JC, et al. Huntingtin exon 1 fibrils feature an interdigitated β-hairpin–based polyglutamine core. *Proceedings of the National Academy of Sciences* **113** (2016) 1546–1551.

[4] Wiegand T, Malär AA, Cadalbert R, Ernst M, Böckmann A, Meier BH. Asparagine and Glutamine Side-Chains and Ladders in HET-s(218–289) Amyloid Fibrils Studied by Fast Magic-Angle Spinning NMR. *Frontiers in Molecular Biosciences* **7** (2020) 582033. doi:10.3389/fmolb.2020.582033.

[5] Perutz MF, Finch JT, Berriman J, Lesk A. Amyloid fibers are water-filled nanotubes. *Proceedings of the National Academy of Sciences* **99** (2002) 5591–5595.

[6] Sikorski P, Atkins E. New model for crystalline polyglutamine assemblies and their connection with amyloid fibrils. *Biomacromolecules* **6** (2005) 425–432.

[7] Maier JA, Martinez C, Kasavajhala K, Wickstrom L, Hauser KE, Simmerling C. ff14sb: improving the accuracy of protein side chain and backbone parameters from ff99sb. *Journal of chemical theory and computation* **11** (2015) 3696–3713.

[8] Robertson MJ, Tirado-Rives J, Jorgensen WL. Improved peptide and protein torsional energetics with the opls-aa force field. *Journal of chemical theory and computation* **11** (2015) 3499–3509.

[9] Hervas R, Rau MJ, Park Y, Zhang W, Murzin AG, Fitzpatrick JAJ, et al. Cryo-EM structure of a neuronal functional amyloid implicated in memory persistence in *Drosophila*. *Science* **367** (2020) 1230–1234. doi:10.1126/science.aba3526.

[10] Kajava AV, Baxa U, Steven AC. Beta arcades: recurring motifs in naturally occurring and disease-related amyloid fibrils. *FASEB J* **24** (2010) 1311–1319. doi:10.1096/fj.09-145979.
