# Supplementary material for: Integrative determination of atomic structure of mutant huntingtin exon 1 fibrils implicated in Huntington disease
Source: Nat Commun. 2024 Dec 30;15:10793. doi: 10.1038/s41467-024-55062-8 (PMC11686214; doi:10.1038/s41467-024-55062-8)
Supplement: Supplementary file 2 — Description of Additional Supplementary Files [file 41467_2024_55062_MOESM2_ESM.pdf]

Description of Additional Supplementary Files

Supplementary Movie 1 - Movie illustrating key features and perspectives for the final fibril architecture for Q44-HTTex1 fibrils. The movie starts with the end view along the z-axis: representing a view along the fibril long axis on the fibril core and flanking segments. Then, a top view is given, and a single b-sheet layer highlighted in context of the fibril. Next, a zoomed view offers a close-up of the side chains in the polyQ fibril core. After zooming out again, the movie shows the b-sheet layer in isolation, with a color code identifying the two polyQ conformers (types "a" and "b") in the fibril core, as well as the flanking segments. The final part of the movie illustrates the structural variations in the flanking segments, which are different in different protein monomers.
